# RNA sequencing dataset describing transcriptional changes in cervical dorsal root ganglia after bilateral pyramidotomy and forelimb intramuscular gene therapy with an adeno-associated viral vector encoding human neurotrophin-3

**DOI:** 10.1016/j.dib.2018.09.099

**Published:** 2018-10-03

**Authors:** Claudia Kathe, Lawrence D.F. Moon

**Affiliations:** a Neurorestoration Group, Wolfson Centre for Age Related Diseases, King׳s College, London, UK; b Center for Neuroprosthetics, Brain Mind Institute, Campus Biotech, École Polytechnique Fédérale de Lausanne, Geneva, Switzerland

## Abstract

Unilateral or bilateral corticospinal tract injury in the medullary pyramids in adult rats causes anatomical and physiological changes in proprioceptive neurons projecting to the cervical spinal cord accompanied by hyperreflexia and abnormal behavioural movements including spasms. In a previous publication, we showed that “Intramuscular Neurotrophin-3 normalizes low threshold spinal reflexes, reduces spasms and improves mobility after bilateral corticospinal tract injury in rats” (Kathe et al., 2016) [1]. We hypothesize that neurotrophin-3 induces these changes by modifying gene expression in affected cervical dorsal root ganglia (DRG). Therefore in this data article, we analyzed the transcriptomes of cervical DRGs obtained during that previous study from naïve rats and from rats after bilateral pyramidotomy (bPYX) with unilateral intramuscular injections of either AAV1-CMV-NT3 or AAV1-CMV-EGFP applied 24 h after injury (Kathe et al., 2016) [1]. A bioinformatic analysis enabled us to identify genes that are likely to be expressed in TrkC+ neurons after injury and which were regulated by neurotrophin-3 in the direction expected from other datasets involving knockout or overexpression of neurotrophin-3. This dataset will help us and others identify genes in sensory neurons whose expression levels are regulated by neurotrophin-3 treatment. This may help identify novel therapeutic targets to improve sensation and movement after neurological injury. Data has been deposited in the Gene Expression Omnibus (GSE82197), http://www.ncbi.nlm.nih.gov/geo/query/acc.cgi?token=avgpicgcjhknzyv&acc=GSE82197.

**Specifications table**

**Table:** 

Subject area	*Biology*
More specific subject area	*Neuroscience*
Type of data	*Figures, tables*
How data was acquired	*RNA sequencing (*Illumina HiSeq. 2500*)*
Data format	Analyzed
Experimental factors	Three groups of rats: 1) bilateral pyramidotomy plus intramuscular injection of AAV1-CMV-NT3; 2) bilateral pyramidotomy plus intramuscular injection of AAV1-CMV-EGFP; 3) naïve, unoperated rats
Experimental features	Analysis of differential expression of poly A RNAs and small RNAs from cervical dorsal root ganglia of three groups of rats 10 weeks after surgery
Data source location	London, UK
Data accessibility	The data is available with this article. All RNAseq data are available in the GEO archive under the accession number GSE82197
	http://www.ncbi.nlm.nih.gov/geo/query/acc.cgi?token=avgpicgcjhknzyv&acc=GSE82197

**Value of the data**

• Our data show that gene expression in cervical dorsal root ganglia was modified by supraspinal injury which is useful because it reveals genes that may underlie maladaptive plasticity causing pain or spasticity. Our data could be compared to datasets from lumbar DRGs that are typically studied in animal models of pain.
• Our data can be used to show that expression levels of some genes in sensory dorsal root ganglia were normalized by intramuscular overexpression of neurotrophin-3. This is valuable because one may seek the overlap between this and related datasets to find genes that are regulated in sensory dorsal root ganglia by neurotrophin-3: some of the gene expression levels changed in the same direction as that predicted by work by other groups.
• This dataset will enable us and others to identify genes that underlie the anatomical and neurophysiological changes in proprioceptive circuits which occur after central nervous system injuries and which may be normalized by treatments. Therapeutic modification of gene expression in sensory ganglia may lead to functional consequences.

## 1 Data

**Fig. 1:**
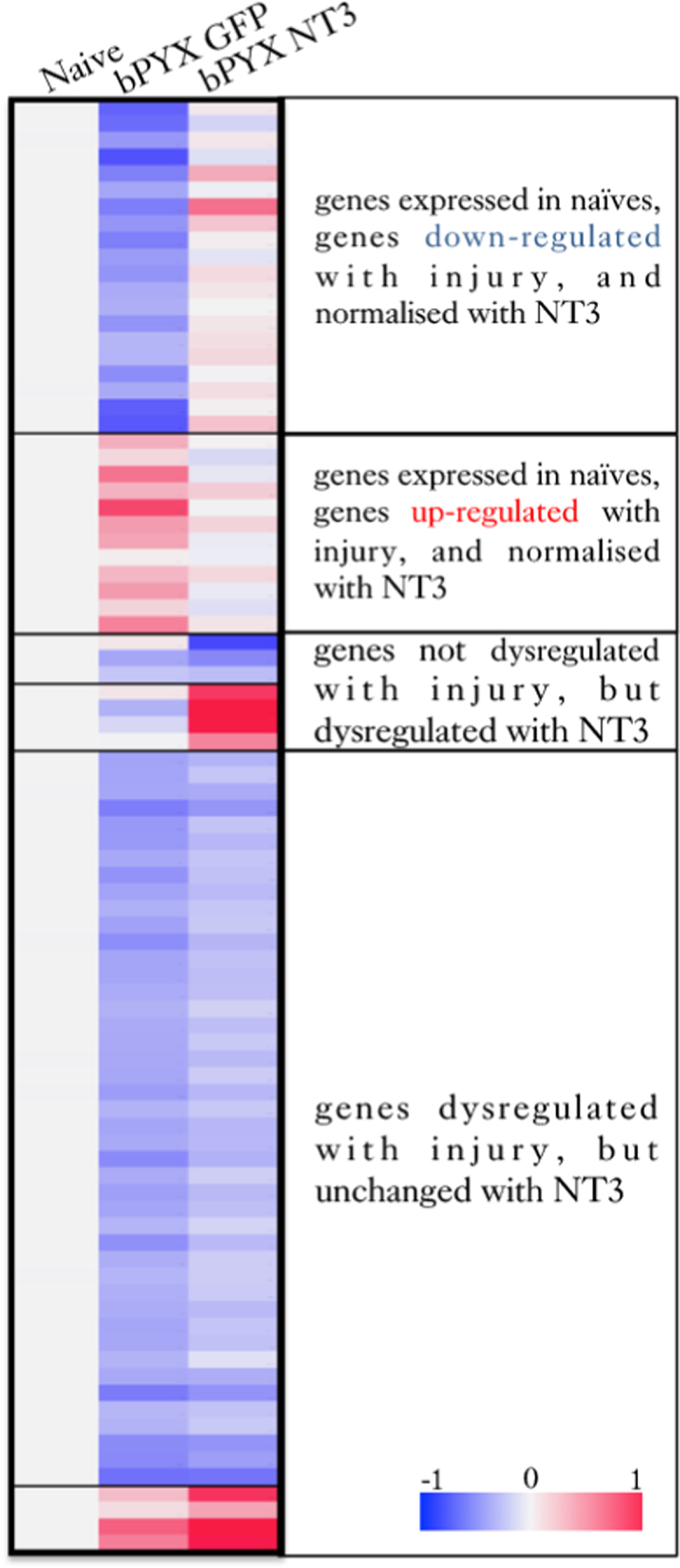
After bilateral transection of the corticospinal tract in the pyramids (bPYX), rats received unilateral intramuscular injections of either AAV1-CMV-NT3 or AAV1-CMV-GFP. Naïve rats were unoperated. Cervical 6–8 DRGs were removed 10 weeks after surgery. RNAseq of poly(A) RNA showed changes in gene expression between these three groups. Lists of poly(A) RNAs for these comparisons are provided in Transparency document, Transparency document.

**Fig. 2:**
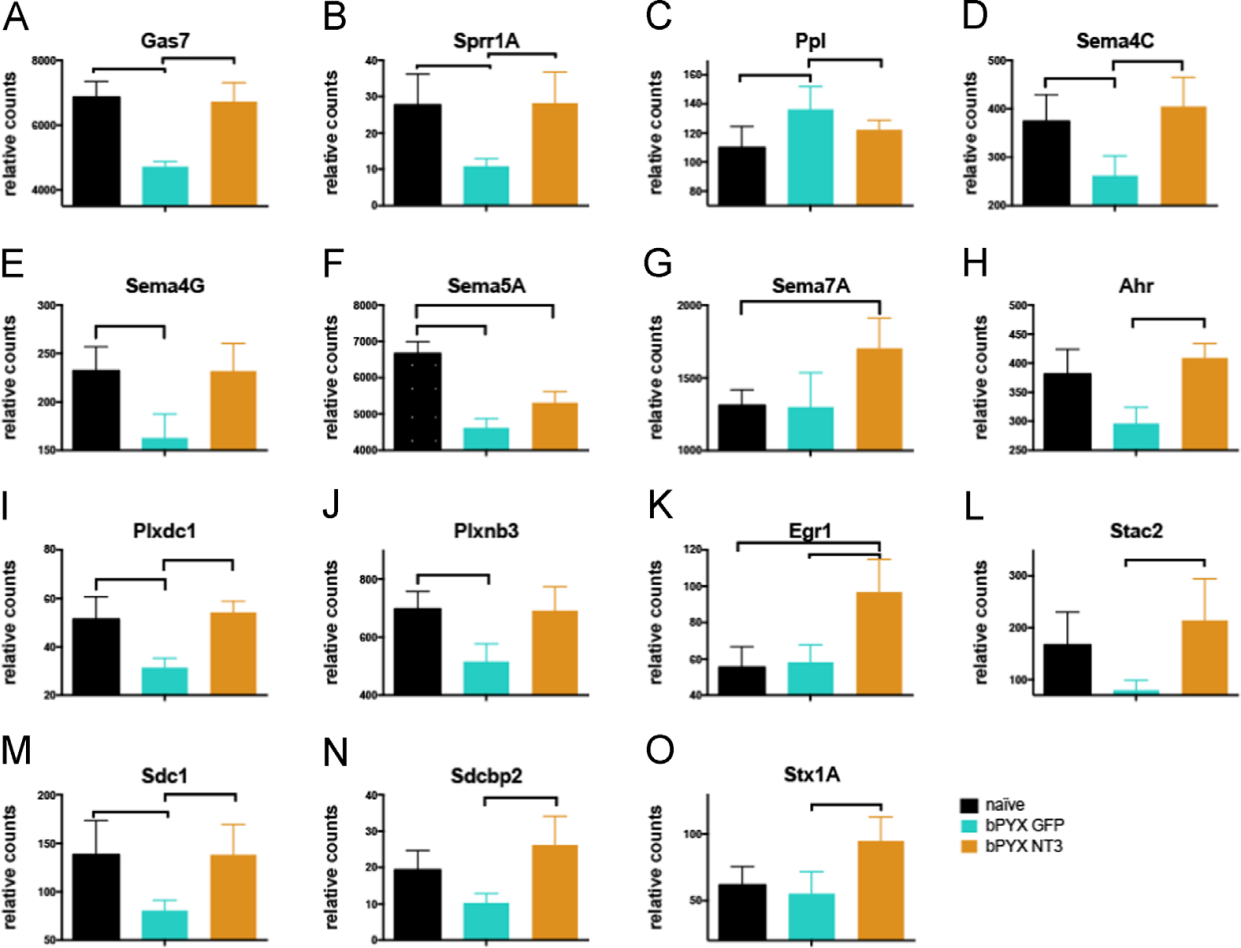
Neurotrophin-3 completely or partially normalizes the levels of many genes in DRG dysregulated by bilateral CST injury. (A)–(O) RNAseq expression values for selected genes, relative counts. (A)–(C) *Gas7*, *Sprr1A* and *Ppl* are important for cytoskeletal reorganization. (D)–(G) *Sema4C*, *Sema4G*, *Sema5A* and *Sema7A* are transmembrane Semaphorins. (H) *Ahr* is a transcription factor regulating Sema4C and Sema7a expression. (I)–(J) *Plxdc1* and *Plxbn3* belong to the group of plexins, which are binding partners for Semaphorins. (K) *Egr1* is a regeneration-associated gene thought to be important for synapse formation. (L)–(O) *Stac2, Sdc1*, *Sdcbp2* and *Stx1A* are synapse associated proteins. *n.b.*, y-axes are not always shown from zero upwards. Means ± SD. Brackets indicate *p* < 0.05.

**Fig. 3:**
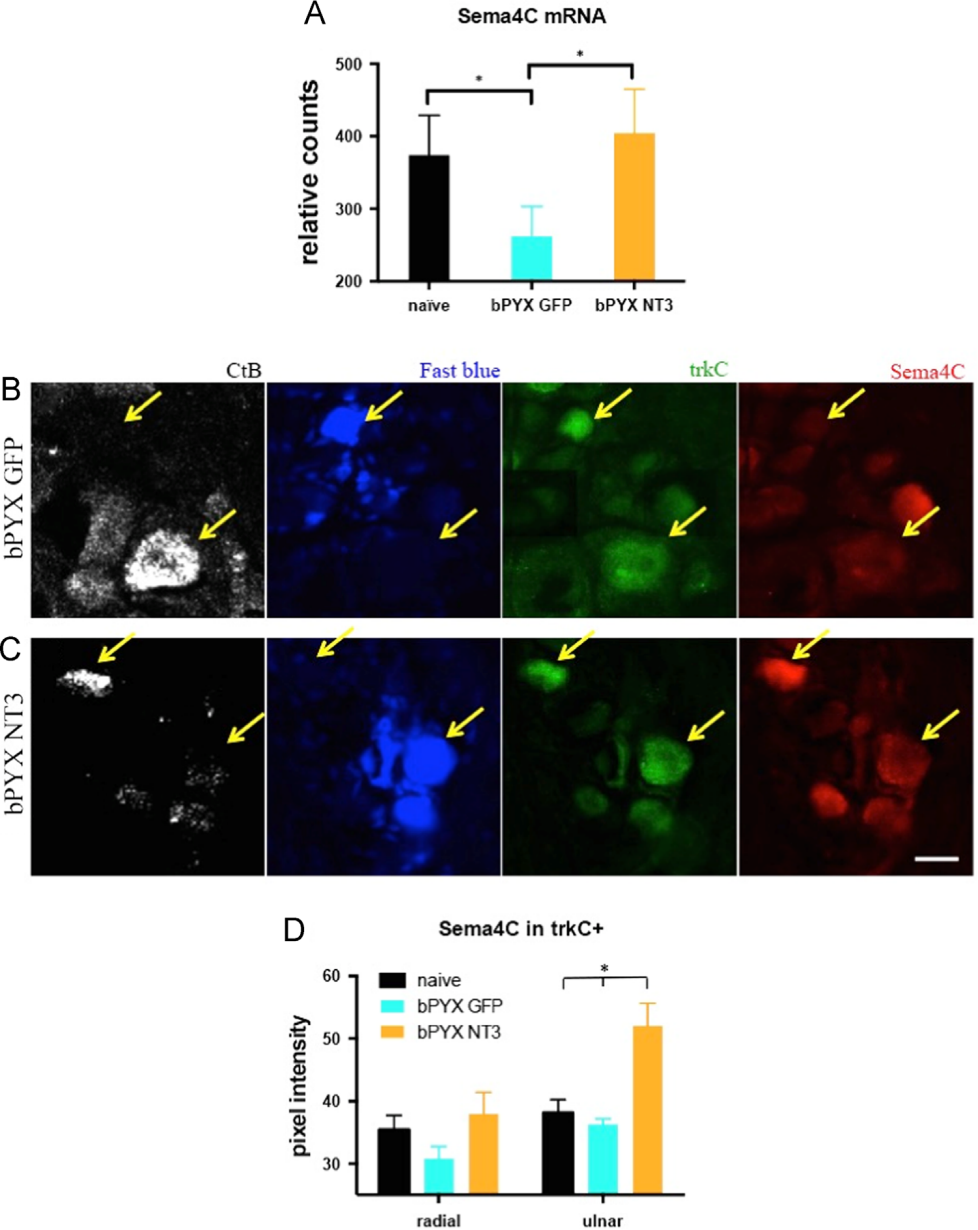
AAV1-CMV-NT3 restores the expression of the axon guidance molecule Sema 4C in cervical DRG neurons. (A) RNAseq showed that *Sema4C mRNA,* a transmembrane axon guidance molecule, was downregulated in bPYX+GFP animals. NT3 normalized Sema4C mRNA levels. (B–C) The ulnar nerve contains afferents from forelimb muscles that were treated with intramuscular AAV-NT3, whereas the radial nerve contains afferents from non-injected muscle groups. Ulnar and radial dorsal root ganglion neurons were traced with CTb and Fast blue respectively. They were immunolabelled with antibodies against TrkC and Sema4C. Yellow arrows indicated CTb^+^ TrkC^+^ and Fast blue^+^ TrkC^+^ example neurons, which were analysed for Sema4C staining intensity. (D) Pixel intensity was increased in ulnar nerve TrkC^+^ dorsal root ganglia neurons after neurotrophin-3 treatment (two-way ANOVA, group *F* = 11.5 *p* < 0.001; ulnar nerve DRG neurons bPYX+NT3 vs naive/bPYX+GFP, *p*-values < 0.01). Means ± SDs. Sema4C׳s binding partner Plexin B2 is expressed by spinal motor neurons as well as other spinal neurons (Allen Brain Atlas; adult mouse spinal cord).

These data sets relate to the article entitled “Intramuscular Neurotrophin-3 normalizes low threshold spinal reflexes, reduces spasms and improves mobility after bilateral corticospinal tract injury in rats” [1]. Gene count tables can be found in the Gene Expression Omnibus (GSE82197), http://www.ncbi.nlm.nih.gov/geo/query/acc.cgi?token=avgpicgcjhknzyv&acc=GSE82197. RNA sequencing revealed poly-A RNAs (Supplementary Table 2) and small RNAs (Supplementary Table 3) that were expressed in cervical DRG of unoperated (naïve) adult rats and of corticospinal tract injured rats either treated with intramuscular injections of AAV1-CMV-EGFP (bPYX+GFP) or AAV1-CMV-NT3 (bPYX+NT3). Supplementary Tables 4 and 5 show RNAs that were regulated by bilateral pyramidotomy plus treatments. Some RNAs were dysregulated by injury and stayed dysregulated with neurotrophin-3 (Supplementary Table 6). Some RNAs were dysregulated by neurotrophin-3 and not by injury alone (Supplementary Table 8). Other RNAs were significantly dysregulated by injury and normalized with neurotrophin-3 treatment (Fig. 1, Fig. 2, Supplementary Table 7). For example, *Semaphorin 4C* was down-regulated in DRG after injury and but normalized by NT3 (Fig. 3A); immunolabelling showed that TrkC^+^ neurons with ulnar afferents (*i.e.*, from a treated muscle) had increased Sema4C expression after intramuscular neurotrophin-3 treatment, but not the cell bodies with afferents from the radial nerve (Fig. 3B–D).

To identify genes that might be regulated in TrkC+ neurons by NT3, we analysed a dataset (GSE38074) of mRNAs from TrkC^+^ neurons from postnatal day 0 DRG either of mice with increased muscular neurotrophin-3 (mlcNT3 versus wildtype control) or of mice lacking neurotrophin-3 (on a Bax knockout background to prevent cell death; NT3KO BaxKO versus BaxKO) [2]. We discovered five genes (Table 2) whose levels were higher in our key comparison (bPYX+NT3 vs bPYX+GFP) and also higher in mlcNT3 vs wildtype and lower in NT3KO BaxKO vs BaxKO. In future, this dataset will allow us and others to study gene expression changes related to neuronal plasticity in more detail.

**Table 2:** The levels of five mRNAs correlated with higher NT3 in all three key comparisons: bPYX+NT3 v bPYX+GFP, mlcNT3 vs wildtype and NT3KO BaxKO v BaxKO (*p* < 0.05). Vgf (*n.b.*, not acronymic and not Vegf) is a secreted peptide known to be expressed in sensory ganglia and whose expression is upregulated by neurotrophins [3]. We did not find any genes whose levels correlated negatively with NT3 levels in all three comparisons (i.e., were lower in bPYX+NT3 vs bPYX+GFP and lower in mlcNT3 vs wildtype and higher in NT3KO BaxKO vs BaxKO).

	Log_2_ fold change in bPYX+NT3 vs bPYX+GFP	Log_2_ fold change in mlcNT3 vs wildtype	Log_2_ fold change in NT3KO BaxKO versus BaxKO
Spry4	0.4	2.2	− 1.7
Rasgef1c	0.3	0.4	− 0.6
Rtn4ip1	0.3	0.4	− 0.8
Plk2	0.2	0.6	− 1.2
Vgf	0.5	1.2	− 2.5

## 2 Experimental design, materials and methods

Adult rats received bilateral transection of the corticospinal tracts in the medullary pyramids (bPYX). Twenty-four hours later, rats received unilateral injection into their forelimb flexors of either an Adeno-associated viral vector (AAV1) encoding human neurotrophin-3 (NT3) or Green Fluorescent Protein (GFP). These rats underwent behavioural testing, neurophysiological assessment and nerve tracing and tissues were recovered for histology as described elsewhere [1]. Owing to the fact that we found evidence that intramuscular neurotrophin-3 affects spinal networks via proprioceptive afferents, we decided to investigate transcriptional changes in the ipsilateral cervical DRGs. C6-C8 cervical dorsal root ganglia (DRG) from the treated side were removed ten weeks after transection and snap frozen in liquid nitrogen. DRG were pooled from the treated side, homogenized and Poly(A) RNAs and small RNAs were sequenced separately. All animal experiments were performed after appropriate review by the Animal Welfare Ethical Review Board at King׳s College London and subject to our Home Office Project Licence (70/7865). We isolated RNA from homogenates of DRGs to assess overall gene expression in neurons and glia.

### 2.1 Total RNA extraction for sequencing

Total RNA for sequencing was extracted from C6-C8 DRGs which were pooled unilaterally from the treated side per animal (and not between animals). Tissue was homogenized in QIAZOL (Qiagen, 79306) and nucleic acids were separated in Phase Locked Gel columns (5Prime, 230 2830). 1.5 volumes of 100% ethanol were added to the aqueous phase and transferred to filtered spin columns from the extraction kit for poly A RNA and small RNAs (miRNeasy kit, Qiagen, 217004) and we proceeded according to manufacturer׳s instructions. Samples were also DNase I treated with double volume of the recommended amount (Qiagen, 79254). We estimated the total RNA quality and quantity by spectrophotometry (NanoDrop, ND-1000) and measured the RNA integrity numbers (RINs) (Agilent RNA 6000 Nano Reagents Part I and Agilent 2100 Bioanalyzer). The RIN for each sample was greater than 7.6, with an average of 8 (Supplementary Table 1).

### 2.2 Poly A RNA sequencing and analysis

The samples were subjected to poly(A) enrichment with oligo-dT beads (Illumina) and the library was prepared using the TruSeq Stranded prep kit (Illumina). Samples were run as a barcoded multiplex over 4 lanes on the Illumina HiSeq. 2500 platform using the Rapid SBS kit v2 (50 bp read length, paired end). Illumina universal paired end adapters were used:

5׳ P-**GATCGGAAGAGC**GGTTCAGCAGGAATGCCGAG.

5׳ ACACTCTTTCCCTACACGAC**GCTCTTCCGATC**T.

The success of the sequencing run and quality of the raw data were assessed by a range of metrics using custom scripts. The 50 bp paired-end reads were aligned to the Rattus norvegicus reference genome (Rnor_6.0) using TopHat2 with default parameters (except for setting mate-inner-dist = 100 and mate-std-dev = 50). Just over 30 million read-pairs per sample were obtained on average (mean ± SD, 32.1 ± 4.4 million) and around 94% of these could be aligned to the reference genome. Duplicate reads were identified using Picard Tools MarkDuplicates (Picard Tools by The Broad Institute) and the highest quality read at each position was retained. Reads mapping to each gene feature were counted using HTseq to create a raw gene count table for 15,075 RefSeq annotated genes with at least one mapped read (Supplementary Table 2). Due to a relatively large number of reads annotated as ‘no feature’ (i.e. mapping to intronic or intergenic regions) and exclusion of duplicate reads and those mapping to multiple locations, the total number of read-pairs mapped to gene features per sample was 12.8 ± 1.8 million (mean ± SD). Further quality control plots and exploratory analyses including principal component analysis (PCA) were performed to assess the overall behaviour of the dataset. Although minor outliers were observed, all samples were included for subsequent analysis for mRNA expression profiles. A detection filter requiring > 10 reads on average in at least 6 samples was applied and 11,528 genes were considered expressed and retained for differential expression analysis using the EdgeR package comparing and creating the following three data sets: bPYX+GFP vs naive, bPYX+NT3 vs bPYX+GFP, bPYX+NT3 vs naive. Lists of differentially regulated poly(A) RNAs were based on *p* < 0.05; changes in expression level (log_2_ fold change) for all poly(A) RNAs (i.e., whether significantly regulated or not) for all three comparisons is provided in Supplementary Table 4.

### 2.3 Small RNA sequencing and analysis

Libraries were prepared using the Small Library Prep Set for Illumina (NEBNext, multiplex compatible; NEB) using custom index primers. Samples were run as a barcoded multiplex over 4 lanes on the Illumina HiSeq. 2500 platform using the Rapid SBS kit v2 (50 bp read length, single end).

miRNA data was mapped to RNor_5.0, in the absence of a miRNA resource for rn6 at that time. The total number of reads was around 13 million (Mean: 13.5 M, SD: 1.7 M). Of those, 96.7% mapped to the target regions and 93.5% mapped to miRNA features. For miRNA sequencing, the analysis pipeline included adapter trimming prior to mapping, and used the short-read aligner Bowtie2. The count table (Supplementary Table 3) was generated with HTSeq-count. RNA sequencing analyses including QC and exclusion of samples from analysis was performed by an impartial third party bioinformatician: one outlier (rat #41) was identified in the QC plots and excluded for further analysis. A detection filter requiring > 10 reads on average in at least 6 samples was applied and 363 small RNAs were considered expressed and retained for differential expression analysis using the EdgeR package comparing and creating following data sets: bPYX+GFP vs naive, bPYX+NT3 vs bPYX+GFP, bPYX+NT3 vs naive. Lists of differentially regulated small RNAs were based on *p* < 0.05. Changes in expression level (log_2_ fold change) for all small RNAs (*i.e.*, whether significantly regulated or not) for all three comparisons is provided in Supplementary Table 5.

### 2.4 Bioinformatic analyses

Next, three lists of poly(A) RNAs were created that were differentially regulated in two of our comparisons. This revealed genes that were 1) dysregulated after injury and then normalized with NT3 treatment, 2) dysregulated after injury and not normalised with NT3 treatment, 3) not dysregulated after injury, but dysregulated with NT3 treatment (Supplementary Tables 6-8).

To identify genes that might be regulated in TrkC+ neurons by NT3 in our experiment, we next took advantage of a publicly available microarray dataset (GSE38074) containing information about expression of mRNAs (but lacking information about small RNAs) in fluorescence-activated cell sorted TrkC^+^ neurons from the postnatal day 0 DRG either of mice with increased muscular NT-3 (mlcNT3 versus wildtype control; *n* = 2/group) or of mice lacking NT-3 (on a Bax knockout background to prevent cell death during development; NT3KO BaxKO versus BaxKO; *n* = 2/group) [2]. We sought genes whose levels correlated positively with NT3 levels in these two comparisons and in bPYX+NT3 versus bPYX+GFP.

### 2.5 Immunofluorescence staining

Tissues were obtained from a previous study [1]. We evaluated with immunolabeling (Table 1) whether neurotrophin-3 treatment regulates Sema4C expression specifically in TrkC^+^ afferent neurons, which include proprioceptive afferents. We distinguished between ulnar and radial afferents by retrograde tracing with Cholera toxin beta (Ctb) subunit and Fast blue respectively (Fig. 3B, C).

**Table 1:** Table showing primary and secondary antibodies.

Antibody	Supplier and Cat number	Concentration
Goat anti-Sema4C	Santa Cruz sc-169282	1:200
Rabbit anti-TrkC	CST 3376	1:1600
Goat anti-CtB	List Laboratories 703	1:2000
Alexa 488 donkey anti-goat	Life Technologies A11055	1:1000
Alexa 488 donkey anti-rabbit	Life Technologies A21206	1:1000
Alexa 594 goat anti-rabbit	Life Technologies A11012	1:1000
DyLight 650 donkey anti-goat	Abcam ab96938	1:1000

Immediately after performing nerve neurophysiology, rats were transcardially perfused with PBS pH 7.4 and tissues were dissected rapidly. Tissue for sectioning on the cryostat was post-fixed by immersion in 4% paraformaldehyde in PBS pH 7.4 overnight and cryoprotected in 30% sucrose in PBS pH 7.4. Tissue was frozen and embedded in O.C.T., DRGs were sectioned transversely at 10 μm thickness respectively and directly mounted onto glass slides. Immunofluorescence staining was performed [1] using antibodies shown below (Table 1). All antibodies were verified by their manufacturer. The Sema4C antibody is raised against a peptide mapping with a C-terminal cytoplasmic domain of Sema4C human origin.

### 2.6 Image analysis

Image analysis was performed with Image J or Zen Imaging software. Pixel intensity of Sema4C staining was analysed in dorsal root ganglia neurons, which contained afferents from ulnar (CTb positive) and radial (Fast blue positive) nerve. The pixel intensity of the cytoplasm of TrkC^+^ neurons was measured. 8 neurons per section and 3 sections per animal were analysed. The mean number per animals was calculated. The mean was averaged within the groups.

## Footnotes

Transparency document Transparency document associated with this article can be found in the online version at https://doi.org/10.1016/j.dib.2018.09.099.
