# Supplementary material for: RNA sequencing dataset describing transcriptional changes in cervical dorsal root ganglia after bilateral pyramidotomy and forelimb intramuscular gene therapy with an adeno-associated viral vector encoding human neurotrophin-3
Source: Data Brief. 2018 Oct 3;21:377–85. doi: 10.1016/j.dib.2018.09.099 (PMC6197729; doi:10.1016/j.dib.2018.09.099)
Supplement: Supplementary file 2 — Supplementary material [file mmc2.docx]

## Supplementary Tables

| 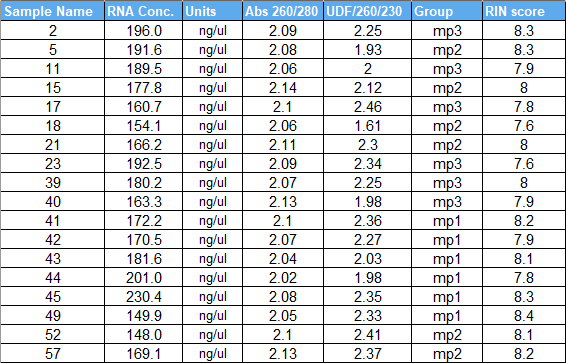  Supplementary Table 1: Table showing sample identification numbers submitted for RNA sequencing together with sample concentration (ng/ul), ratio of absorbance readings at 260nm/280nm and at 260nm/230nm, RIN score. Group mp1 = naïve; Group mp2 = bPYX+GFP; Group mp3 = bPYX+NT3. |
| --- |

Supplementary Table 2: Excel file containing count data for 15,075 poly(A) RNAs in cervical sensory ganglia with at least one mapped read. Sample names are as shown in Supplementary Table 1. Group means and standard deviations are shown.

Supplementary Table 3: Excel file showing counts per sample for 753 small RNAs. Sample names are as shown in Supplementary Table 1. Group means and standard deviations are shown for all samples and also when outlier sample #41 was omitted.

Supplementary Table 4: Excel file containing data for poly(A) RNAs in cervical sensory ganglia. Three comparisons are shown (one per worksheet); bPYX+GFP *versus* naïve; bPYX+NT3 *versus* naïve; bPYX+NT3 *versus* bPYX+GFP. Each list shows all poly(A) RNAs above the cut-off threshold, whether or not significantly regulated. Log_2_ fold change is positive when the first named group in that worksheet has higher expression level than the second named group in that worksheet (e.g., bPYX+NT3 *versus* bPYX+GFP).

Supplementary Table 5: Excel file containing data for small RNAs in cervical sensory ganglia. Three comparisons are shown (one per worksheet); bPYX+GFP *versus* naïve; bPYX+NT3 *versus* naïve; bPYX+ NT3 *versus* bPYX+GFP. Each list shows all small RNAs above the cut-off threshold, whether or not significantly regulated. Table shows miRNA name, log_2_ fold change, log CPM, LR, PValue and FDR. Log_2_ fold change is positive when the first named group in that worksheet has higher expression level than the second named group in that worksheet (e.g., bPYX+NT3 *versus* bPYX+GFP). Sample #41 was omitted as it was an outlier.
