# Supplementary material for: RNA sequencing dataset describing transcriptional changes in cervical dorsal root ganglia after bilateral pyramidotomy and forelimb intramuscular gene therapy with an adeno-associated viral vector encoding human neurotrophin-3
Source: Data Brief. 2018 Oct 3;21:377–85. doi: 10.1016/j.dib.2018.09.099 (PMC6197729; doi:10.1016/j.dib.2018.09.099)
Supplement: Supplementary file 7 — Supplementary material [file mmc7.docx]

| Transcript name | Log_2_ fold change  (bPYX+GFP vs naïve) | Log_2_ fold change  (bPYX+NT3 vs naïve) |
| --- | --- | --- |
| Abcg2 | -0.37 | -0.46 |
| Aif1l | -0.39 | -0.37 |
| Aldh1a1 | -0.34 | -0.51 |
| B3gnt5 | -0.74 | -0.74 |
| Ccnd1 | -0.48 | -0.36 |
| Cntf | -0.47 | -0.46 |
| Col14a1 | -0.35 | -0.34 |
| Col1a1 | -0.50 | -0.34 |
| Col3a1 | -0.35 | -0.37 |
| Col5a2 | -0.26 | -0.32 |
| Dll4 | 0.52 | 0.80 |
| Eltd1 | 0.35 | 0.39 |
| Emp2 | -0.40 | -0.32 |
| Fa2h | -0.60 | -0.47 |
| Fam198b | -0.35 | -0.36 |
| Fgl2 | -0.35 | -0.39 |
| Flt4 | 0.85 | 1.14 |
| Hectd2 | -0.30 | -0.38 |
| Igsf11 | -0.27 | -0.28 |
| Il33 | -0.30 | -0.37 |
| Itga6 | -0.32 | -0.31 |
| Itm2a | -0.34 | -0.43 |
| Lect1 | -0.36 | -0.31 |
| Mal | -0.44 | -0.46 |
| Maob | -0.26 | -0.33 |
| Mme | -0.37 | -0.45 |
| Myoc | -0.33 | -0.42 |
| Myom2 | -0.60 | -0.51 |
| P4ha1 | -0.31 | -0.27 |
| Padi2 | -0.43 | -0.44 |
| Peli2 | -0.36 | -0.39 |
| Plekhb1 | -0.24 | -0.26 |
| Pmp2 | -0.60 | -0.46 |
| Pmp22 | -0.31 | -0.31 |
| Rcn1 | -0.23 | -0.28 |
| RGD1565616 | -0.27 | -0.30 |
| Rhobtb3 | -0.30 | -0.45 |
| Secisbp2l | -0.37 | -0.36 |
| Sema5a | -0.34 | -0.37 |
| Slc44a1 | -0.25 | -0.37 |
| Slco1a2 | -0.32 | -0.49 |
| Slco1c1 | -0.76 | -0.76 |
| Stag1 | -0.22 | -0.35 |
| Stc1 | 0.77 | 0.88 |
| Svip | -0.26 | -0.33 |
| Trhr | -0.58 | -0.72 |
| Ugt8 | -0.63 | -0.67 |
| Zbtb16 | -0.94 | -1.08 |
| rno-miR-125a-5p | 0.34 | 0.22 |
| rno-miR-139-5p | -0.21 | -0.3 |
| rno-miR-152-5p | -0.37 | -0.4 |
| rno-miR-182 | 0.21 | 0.22 |
| rno-miR-183-3p | 0.32 | 0.33 |
| rno-miR-193b-3p | -0.3 | -0.34 |
| rno-miR-193b-5p | -0.73 | 0.32 |
| rno-miR-20b-3p | -0.35 | -0.31 |
| rno-miR-219a-1-3p | -0.47 | -0.4 |
| rno-miR-26b-5p | 0.23 | 0.37 |
| rno-miR-328a-3p | 0.24 | 0.3 |
| rno-miR-361-3p | -0.31 | -0.32 |
| rno-miR-363-5p | -0.62 | -0.63 |
| rno-miR-370-3p | -0.39 | -0.37 |
| rno-miR-493-3p | -0.23 | -0.36 |
| rno-miR-496-3p | 0.49 | 0.46 |
| rno-miR-676 | -0.3 | -0.33 |
| rno-miR-770-5p | 0.77 | 0.59 |

Supplementary Table 6: Sequencing identified 48 mRNAs and 18 small RNAs in cervical sensory ganglia whose expression levels were modified by bPYX+GFP *versus* the naïve group (p<0.05) and not by bPYX+NT3 *versus* bPYX+GFP (p>0.05).
