# Supplementary material for: RNA sequencing dataset describing transcriptional changes in cervical dorsal root ganglia after bilateral pyramidotomy and forelimb intramuscular gene therapy with an adeno-associated viral vector encoding human neurotrophin-3
Source: Data Brief. 2018 Oct 3;21:377–85. doi: 10.1016/j.dib.2018.09.099 (PMC6197729; doi:10.1016/j.dib.2018.09.099)
Supplement: Supplementary file 8 — Supplementary material [file mmc8.docx]

­­­

| Transcript name | Log_2_ fold change  (bPYX+GFP vs naïve) | log_2_ fold change  (bPYX+NT3 vs bPYX+GFP) |
| --- | --- | --- |
| Abca4 | 0.55 | -0.57 |
| Arg1 | -1.01 | 1.10 |
| Aurkb | -0.94 | 0.73 |
| Cdkn1a | -0.45 | 0.49 |
| Cldn11 | 0.36 | -0.53 |
| Crisp1 | -1.28 | 1.20 |
| Cxcl14 | -0.71 | 1.01 |
| Fmo3 | 0.78 | -0.79 |
| Frzb | 0.54 | -0.36 |
| Gas7 | -0.35 | 0.26 |
| Gfap | -0.70 | 1.26 |
| Gpnmb | 0.86 | -0.89 |
| Id1 | 0.60 | -0.45 |
| Loxl4 | -0.51 | 0.72 |
| Ly6g6e | -0.74 | 0.70 |
| Myo10 | -0.45 | 0.33 |
| Myof | 0.55 | -0.62 |
| Net1 | 0.23 | -0.28 |
| Nfil3 | -0.51 | 0.60 |
| Pdia5 | -0.32 | 0.35 |
| Plk2 | -0.29 | 0.25 |
| Plxdc1 | -0.49 | 0.56 |
| Ppl | 0.49 | -0.36 |
| Rasa3 | -0.26 | 0.31 |
| Rcan1 | -0.23 | 0.34 |
| Sdc1 | -0.53 | 0.52 |
| Sema4c | -0.33 | 0.40 |
| Sprr1a | -1.11 | 1.13 |
| Tgm1 | -1.21 | 1.45 |
| Upk1b | 0.64 | -0.68 |
| Zic2 | 0.38 | -0.51 |
| Zic5 | 0.73 | -0.66 |
| rno-let-7b-3p | 0.25 | -0.3 |
| rno-miR-145-5p | -0.45 | 0.35 |
| rno-miR-146b-5p | -0.6 | 0.36 |
| rno-miR-148a-3p | 0.77 | -0.66 |
| rno-miR-148b-3p | 0.63 | -0.61 |
| rno-miR-30d-3p | -0.4 | 0.31 |
| rno-miR-30e-3p | 0.45 | -0.26 |
| rno-miR-344b-1-3p | -0.53 | 0.34 |
| rno-miR-490-3p | -0.3 | 0.26 |

Supplementary Table 7: Sequencing identified 32 mRNAs and 9 small RNAs in cervical sensory ganglia whose expression levels were modified by pyramidotomy (bPYX+GFP versus naïve, p<0.05) and normalized by intramuscular NT3 treatment relative to pyramidotomy plus GFP (bPYX+NT3 versus bPYX+GFP, p<0.05).
