# Supplementary material for: RNA sequencing dataset describing transcriptional changes in cervical dorsal root ganglia after bilateral pyramidotomy and forelimb intramuscular gene therapy with an adeno-associated viral vector encoding human neurotrophin-3
Source: Data Brief. 2018 Oct 3;21:377–85. doi: 10.1016/j.dib.2018.09.099 (PMC6197729; doi:10.1016/j.dib.2018.09.099)
Supplement: Supplementary file 9 — Supplementary material [file mmc9.docx]

| Transcript name | Log_2_ fold change  (bPYX+NT3 vs bPYX+GFP) | Log_2_ fold change  (bPYX+NT3 vs naïve) |
| --- | --- | --- |
| Adipoq | -2.11 | -1.85 |
| Cpa3 | -0.53 | -0.88 |
| Egr1 | 0.49 | 0.76 |
| Mall | 1.15 | 0.90 |
| Map3k14 | 0.86 | 0.87 |
| Ncan | 0.31 | 0.48 |
| Prex2 | -0.28 | -0.38 |
| rno-miR-181c-5p | 0.53 | 0.5 |
| rno-miR-582-5p | 0.71 | 0.57 |
| rno-miR-193a-3p | 1.66 | 1.76 |
| rno-miR-137-3p | 0.4 | 0.71 |
| rno-miR-205 | 0.44 | 0.45 |
| rno-miR-22-3p | 0.45 | 0.36 |
| rno-miR-29a-5p | 0.64 | 0.55 |

Supplementary Table 8: Sequencing identified seven mRNAs and seven small RNAs in cervical sensory ganglia whose expression levels were modified by bPYX+NT3 *versus* bPYX+GFP (p<0.05) or bPYX+NT3 *versus* naïve (p<0.05) and not in bPYX+GFP versus naïve (p>0.05).
